# Effect of Acetonitrile on the Conformation of Bovine Serum Albumin

**DOI:** 10.1021/acsomega.4c07274

**Published:** 2024-11-21

**Authors:** Samal Kaumbekova, Masatake Sugita, Naoya Sakaguchi, Yuta Takahashi, Akira Sadakane, Masakazu Umezawa

**Affiliations:** 1 Department of Medical and Robotic Engineering Design, Faculty of Advanced Engineering, Tokyo University of Science, 6-3-1 Niijuku, Katsushika, Tokyo 125-8585, Japan; 2 Department of Computer Science, School of Computing, Institute of Science Tokyo, Tokyo 152-8552, Japan; 3 Middle Molecule IT-based Drug Discovery Laboratory (MIDL), Institute of Science Tokyo, W8-76, 2-12-1 Ookayama, Meguro-ku, Tokyo 152-8550, Japan; 4 Department of Materials Science and Technology, Faculty of Advanced Engineering, Tokyo University of Science, 6-3-1 Niijuku, Katsushika, Tokyo 125-8585, Japan

## Abstract

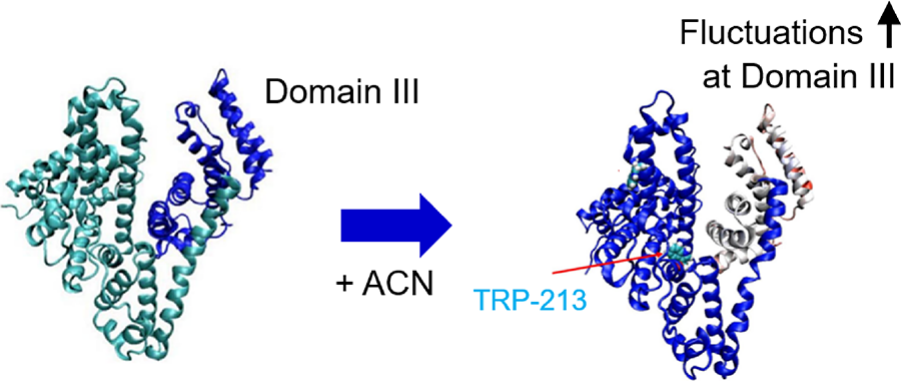

The use of organic solvents in drug delivery systems (DDSs) either to produce albumin nanoparticles or to manipulate the binding of target molecules to albumin, a promising nanocarrier material, presents challenges due to the conformational changes induced in the protein. In this study, we investigated the alterations in the conformation of bovine serum albumin (BSA) caused by acetonitrile (ACN) in aqueous solution by using a combination of spectroscopic analysis and molecular dynamics (MD) simulations. Ultraviolet (UV) absorption, fluorescence, and infrared (IR) absorption spectroscopy were used to analyze the BSA conformation in the solutions containing 0–60 vol % ACN. Additionally, MD simulations were conducted to elucidate the interactions between BSA and solvent components, focusing on the structural changes in the hydrophobic pocket with Trp residues of the albumin. Increasing the ACN concentration leads to significant changes in the BSA conformation, as evidenced by shifts in UV fluorescence wavelength, decreased intensity, and alterations in IR absorption bands. Furthermore, the formation of protein aggregates was observed at high ACN concentration (30 vol % ACN), shown by increased hydrodynamic diameter distribution. MD simulations further demonstrate that the presence of ACN molecules near the hydrophobic pocket with the Trp-213 residue increases the fluctuations in the positions of amino acids observed near the hydrophobic pocket with Trp-213. Moreover, the intrusion of water molecules into the hydrophobic pocket under 60% ACN conditions with highly decreased solvent polarity was correlated with the changes in the BSA secondary structure. These findings enhance our understanding of how solvent polarity affects the albumin conformation, which is crucial for optimizing albumin-based DDS applications.

## Introduction

The drug delivery system (DDS) functions to efficiently administer and protectively transport biomolecules, such as poorly soluble drugs, proteins, and gene therapy substances, to targets in living tissue by encapsulating them inside.^1^ Conventional nanocarriers have challenges that limit targeted drug delivery, including nonspecific uptake into phagocytes and off-target distribution, nonspecific immune activation, poorly controlled drug release in vivo, and poor intracellular uptake, depending on their structure.^2^ Moreover, the good biocompatibility and biodegradability of DDS are essential to avoid the toxicological effects of nanocarriers.^3^ In this context, protein-based nanocarriers have attracted attention as biocompatible colloidal nanomaterials with various structural designs for improved functionality of DDS.^4^^,^^5^ Proteins can load drugs based on different noncovalent interactions within protein structures and between various amino acid residues and drugs.^6,7^

The desolvation procedure is one of the conventional methods to prepare protein-based drug carriers with predictable size in the presence of water-soluble organic solvents used as desolvating agents.^8,9^ Among different organic solvents, acetonitrile (ACN) is widely used in the pharmaceutical industry due to its low freezing/boiling points, comparatively low toxicity, low viscosity, and great solvation ability for polar and nonpolar solutes.^10^ The addition of ACN can cause biomacromolecules with hydrophilic surfaces to aggregate and eventually precipitate by reducing the specific inductivity of water and bringing dehydration.^11^ As a result, ACN might be used as an antisolvent for protein desolvation and the formation of protein nanoparticles by precipitation.^12^

Albumin is the most abundant protein in the blood and has roles in controlling osmotic pressure and transporting substances in blood vessels, with possible applications in controlled drug delivery.^13,14^ The protein is characterized by high stability, high solubility in aqueous solutions, and the ability to bind lipophilic molecules.^14^ Albumin is known for transporting and distributing hormones, steroids, fatty acids, endogenous ligands, and metabolites in vivo.^15−17^ Furthermore, a wide variety of molecules and substances, including drugs, genes, peptides, vaccines, and antibodies, can bind to albumin.^18^ Its nontoxic and nonimmunogenic abilities, as well as its long circulatory half-life, are also the reasons albumin has attracted attention as a drug carrier.^16,19^

Albumin has a molecular weight of approximately 66–67 kDa with 583–585 amino acid residues in its structure depending on the type of species to which albumin belongs to. The protein monomer is composed of three domains (I, II, and III) and six subdomains (IA, IB, IIA, IIB, IIIA, and IIIB).^20^ Among these, subdomains IIA and IIIA are the drug-binding sites, called Sudlow sites I and II, respectively.^21^ Previous studies have elucidated the characteristics of the protein binding sites and amino acid residues involved in drug binding.^22,23^ For example, a molecular dynamics (MD) study showed the possible mechanism of binding of dalbavancin, a hydrophobic drug, to a hydrophobic pocket in subdomain IA of human serum albumin (HSA), causing conformational changes in surrounding amino acid residues, such as phenylalanine Phe-70.^22^

Although organic solvents are widely used in the synthesis of DDS, the conformational changes that cause cosolvent-induced denaturation of proteins are an important subject in biochemistry and biophysics studies.^24−26^ Moreover, the solvent polarity is important in the context of the interactions between protein and solvent.^27^ For example, the unfolding of β-lactoglobulin and protein aggregation in the presence of ethanol is associated with the altered intramolecular hydrophobic interactions under reduced solvent polarity conditions.^28^ Similarly, bovine serum albumin (BSA) is fully folded in an aqueous solution but unfolds in low concentrations of dimethyl sulfoxide (DMSO) and aggregates in the presence of higher concentrations of DMSO.^24^

Furthermore, the effect of the organic solvents on the drug-loading sites of albumin is of high interest considering the importance of the stability of the drug-loading sites. BSA has two tryptophan (Trp) residues, Trp-134 and Trp-213, located in two different subdomains. Trp-134 is more exposed to the solvent as it is located on the surface of BSA in hydrophilic subdomain IB. In comparison, Trp-213 is located in hydrophobic subdomain IIA, which is important for drug binding. As suggested by previous studies, the microenvironment around Trp residues is important for understanding the mechanisms underlying the conformational changes of BSA. In particular, Trp fluorescence analysis is commonly used to study the changes in the microenvironment of the Trp residues and the albumin structural conformation.^13^ For example, the binding of halothane, an anesthetic compound, to serum albumin was studied, suggesting that halothane binds to the hydrophobic domain with the Trp residues, as was observed by decreased Trp fluorescence intensity.^29^ Moreover, the microenvironment around Trp residues is closely related to structural changes, suggesting, for example, loss of tertiary structure of BSA owing to DMSO-induced microenvironmental changes around Trp residues.^24^ Although the effect of several solvents on the albumin conformation and Trp fluorescence emission has been investigated, the effects of solvent polarity on the hydrophobic drug-binding pocket with Trp-213 have still not been clarified.

Although ACN is a highly volatile organic solvent, considering the wide applications of ACN in the pharmaceutical industry and DDS, in this study, the impact of solvent polarity on the albumin conformation was investigated using a mixture of water and ACN, which is less polar than water. The solvent compositions were varied from 0 to 60 vol % ACN. Ultraviolet (UV) fluorescence spectroscopy and infrared (IR) absorption spectroscopy were used to investigate the structural changes in the BSA conformation. Hydrodynamic diameter distribution and ζ potential were studied to characterize the possible formation of protein aggregates at different solvent polarities. Furthermore, MD simulations were performed to study the interactions between Trp residues and solvent components and to elucidate the changes in the microenvironment of the hydrophobic pocket of albumin in the presence of ACN at different concentrations. The results of this combined experimental and computational study contribute to the understanding of albumin structural changes in the presence of solvents of different polarities, which is essential for the applications of albumin-based DDS.

## Materials and Methods

### Materials

BSA (accession code: UniProtKB entry P02769) and deuterium oxide (D_2_O) were purchased from Sigma-Aldrich Co. (St. Louis, MO, USA). Acetonitrile was purchased from Fujifilm Wako Pure Chemical Co. (Osaka, Japan). All the reagents were used without further purification.

### Ultraviolet Absorption and Fluorescence Spectroscopy

UV absorption of samples was analyzed by using a semimicro quartz glass cuvette (path length, 10 mm; Q-4 Sansyo Co., Ltd., Tokyo, Japan) and a UV–visible–near-infrared spectrometer V770 (Shimadzu Co., Kyoto, Japan). UV fluorescence spectra were analyzed by spectroscopy under irradiation of light of a xenon lamp equipped with a monochromator (RF-5300, Shimadzu).

### Thioflavin T (ThT) Analysis

A 24 μM BSA solution and 40 μM thioflavin T (ThT) were dissolved in a PBS-ACN mixed solution. Three samples were prepared with PBS-ACN ratios of 10:0, 9:1, and 7:3, corresponding to 0, 10, and 30% ACN conditions, respectively. After being stirred in a closed system for 24 h at 25 °C, the samples were left to stand in an open system for 78 h at 25 °C to allow ACN to evaporate. The samples thus obtained were irradiated with 410 nm excitation light, and a fluorescence from 430 to 600 nm was observed.

### Fourier Transform Infrared Spectroscopy

Fourier transform infrared (FT-IR) spectra including amide bands were recorded on an FT-IR-6200 spectrometer (Shimadzu). D_2_O was used as the solvent instead of water because the IR absorption peak at 1600–1650 cm^–1^ derived from the O–H bond in water overlaps the peak of the amide I band and disturbs the analysis. BSA (30 mg/mL) was dissolved in a PBS-ACN (10:0, 9:1, and 7:3) mixed solution and stirred overnight. To avoid ACN evaporation and maintain the different concentrations of organic solvent during incubation with protein, the samples with protein and ACN were tightly closed in vials to achieve sufficient incubation. Each sample (20 μL) was dropped between two CaF_2_ plates with a 25 μm spacer between them, and liquid film FT-IR measurements were performed. The Gaussian fitting of the infrared absorption spectrum of BSA at different conditions was performed to analyze the protein secondary structure using the following absorption peaks: amide II = 1570 cm^–1^, intermolecular extended chains = 1613 cm^–1^, β-sheet = 1628 cm^–1^, α-helix = 1651 cm^–1^, and β-turn = 1677 cm^–1^.

### Hydrodynamic Diameter Distribution, ζ-Potential, and pH Measurements

To characterize the possible formation of protein aggregates, the hydrodynamic diameter distribution, ζ-potential, and pH were measured in the solutions with albumin (3 mg/mL) at different solvent polarities, such as 0, 10, and 30% ACN. The protein and solvents were incubated at 25 °C with constant stirring for 24 h in the presence of 0.15 M NaCl. Hydrodynamic diameter distribution was recorded with polydispersity index (P.I.) by dynamic light scattering (DLS) using an ELSZ-2000ZS (Otsuka Electronics Co., Ltd., Osaka, Japan). The ζ-potential was measured by ELSZ-2000ZZS for samples in specific cells (EZ2305, Otsuka Electronics Co., Ltd., Osaka, Japan), and pH was measured by a pH meter (LAQUA PH-SE, Horiba, Ltd., Japan).

### Molecular Dynamics Simulations

A single BSA monomer structure was simulated in three solvent compositions, such as aqueous solution, 30% ACN, and 60% ACN. Each simulation box consists of a BSA molecule, sodium ion, water, and, in certain cases, ACN. In the case of 0, 30, and 60% ACN, the simulation box contained 0, 2786, and 6207 ACN molecules and 24,392, 18,809, and 11,968 water molecules, respectively. Each simulation box contained 16 sodium ions to neutralize the negative charge of albumin. The initial structure of BSA was obtained from Protein Data Bank (PDB ID: 4f5s, chain A).^20^ The ff14SB^30^ and GAFF force field^31^ was applied to the BSA and ACN molecules, respectively, and the partial charges of ACN were parametrized with the AM1-BCC method.^32^ The TIP3P model^33^ was used as a model of water molecules.

All MD simulations were performed using the graphics processing unit-accelerated PMEMD module (PMEMD.cuda) of the AMBER 20 software package.^34^ The initial structure was minimized for 2000 steps, where the first 1000 steps used the steepest descent method and the remaining 1000 steps used the conjugate gradient method. The systems were then heated from 0 to 300 K within 200 ps with Langevin dynamics in the isothermal–isobaric (NPT) ensemble. The pressure was controlled by using a Berendsen barostat and was maintained at 1 bar. The systems were equilibrated for 10 ns. Production runs were performed based on the replica exchange with the solute tempering (REST) method^35^ under mild conditions. The temperature of BSA for each of the four temperature replicas was set to 300, 305, 310, and 315 K to maintain an exchange rate of approximately 16–20%. Exchanges between adjacent replicas were attempted every 10 ps using the Metropolis scheme. The production runs were performed for 1 μs. It should be noted that we also performed calculations with 8 replicas, where the maximum temperature rose to 335 K. However, in that case, some regions of BSA were significantly disrupted in the 60% ACN condition, so the present study analyzes the results of REST calculations in milder conditions, where the maximum temperature is 315 K.

## Results and Discussion

### UV Absorption

**Figure 1:**
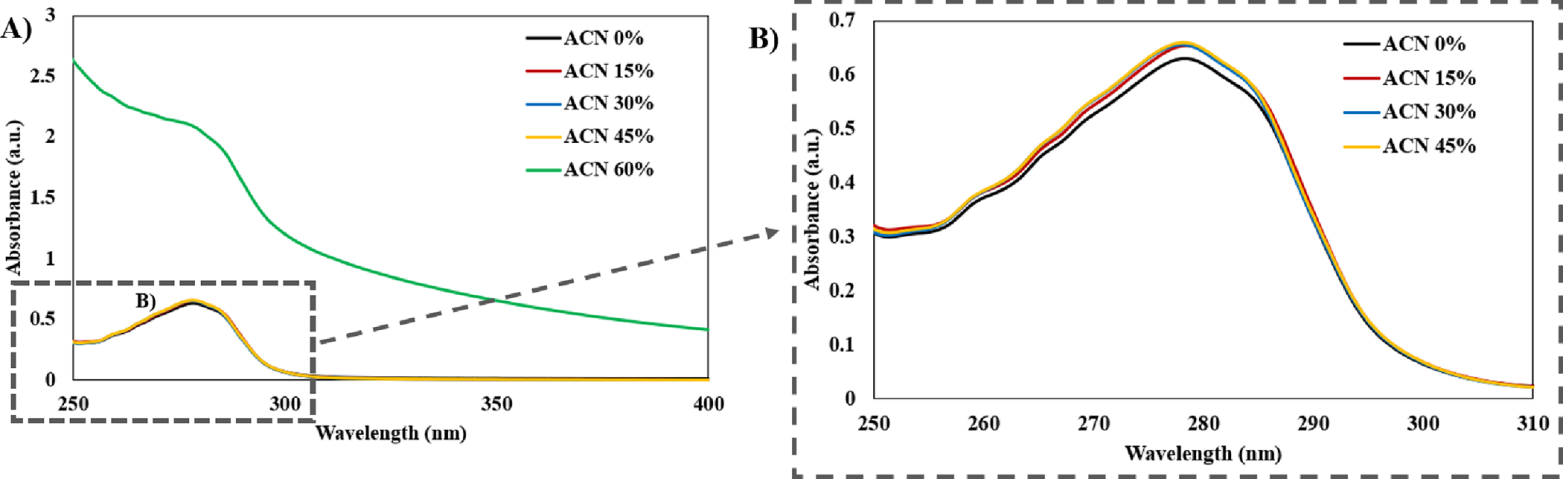
UV absorption spectra of BSA (1 mg/mL) in solvents with different compositions: (A) all samples investigated in this study and (B) enlarged view of 275 nm peaks of the samples.

UV absorption spectra were investigated for aqueous solutions of albumin (1 mg/mL, 0.1% w/v) containing ACN at concentrations of 0, 15, 30, 45, and 60 vol % (Table 1 and Figure 1). Although the majority of the prepared solutions were colorless and transparent, significant cloudiness was observed in the 60% ACN solution. This cloudiness was associated with the denaturation of BSA in the solution with a high concentration of ACN, which led to protein aggregation. According to UV absorption spectral analysis, light scattering was observed in 60% ACN associated with the cloudiness of the solution (Figure 1A). A similar effect of the BSA denaturation and aggregation was observed in the presence of high concentrations of DMSO solvent by Pabbathi et al.^24^ Trp residues, known to form the hydrophobic pocket of BSA, have an absorption peak at a wavelength of around 275 nm. The UV spectra in this study (Figure 1B) showed an increase in the peak around 275 nm in the presence of ACN due to the decreased solvent polarity and microenvironmental change in the hydrophobic pocket.^36^

**Table 1:** Volume and Molar Ratios of the Solvent Components Used in This Study, Aimed to Achieve Different Polarities of the Solvents

solvent	volume ratio		molar ratio		
ACN %	water (vol %)	ACN (vol %)	water (mol %)	ACN (mol %)	ACN concentration (mol/L)
0% ACN	100	0	100	0	0
5% ACN	95	5	98.2	1.8	0.97
10% ACN	90	10	96.3	3.7	1.92
15% ACN	85	15	94.2	5.8	2.86
30% ACN	70	30	87.0	13.0	5.72
45% ACN	55	45	78.0	22.0	8.58
60% ACN	40	60	66.0	34.0	11.44

### UV Fluorescence

**Figure 2:**
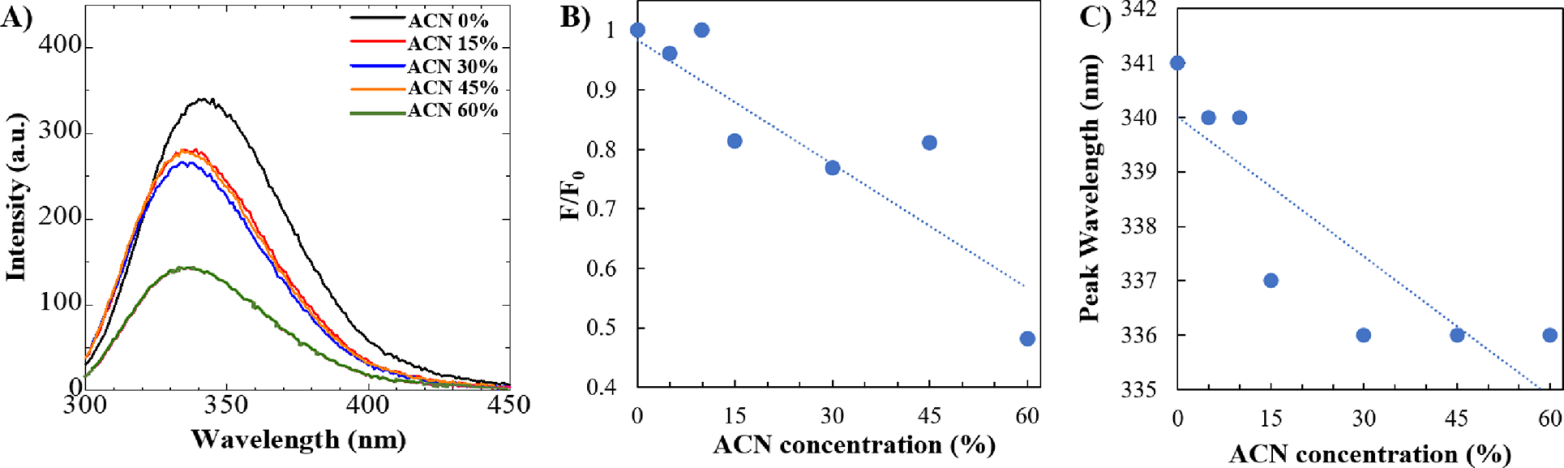
(A) UV fluorescence spectra of BSA (3 mg/mL), (B) intensity ratios, and (C) maximum peak wavelengths in the systems with different solvent compositions and ACN concentrations.

Next, UV fluorescence spectra were investigated for aqueous solutions of albumin (3 mg/mL) containing ACN at concentrations of 0, 5, 10, 15, 30, 45, and 60 vol % (Table 1). As shown in Figure 2A, BSA emits fluorescence at 340 nm under ultraviolet light excitation at wavelengths around 290 nm, consistent with previous studies.^37,38^ According to Figure 2B,C, a blue shift in the fluorescence peak and fluorescence quenching were observed when the ACN concentration exceeded 15 vol %. In particular, the relative fluorescence signal, denoted as the ratio between the final and initial fluorescence emission values (*F*/*F*_0_), was around 1 in the 0–10% ACN solvent conditions (Figure 2B). In comparison, the *F*/*F*_0_ value decreased to 0.8 under the 15–45% ACN solvent conditions. Consistent with our previous observations, 60% ACN significantly affected the protein structure, indicated by the lowest *F*/*F*_0_ value of 0.5. Moreover, a blue shift of 5 nm was observed in the solvent with the ACN concentration exceeding 15 vol %, as shown in Figure 2C. In particular, the peak wavelength decreased from 340 to 341 nm in 0–10% ACN to 336–337 nm in 15–60% ACN solvents. Our results are in good agreement with Liu et al.,^39^ who observed the fluorescence quenching of BSA when the ethanol concentrations exceeded 30% (v/v), while no significant changes were observed when the concentrations of ethanol were less than 20% (v/v). Overall, these observations indicated the conformational changes in the microenvironment around Trp residues with a reduction of the local polarity^38,40^ and increased hydrophobicity around the Trp residues^41,42^ due to the decreased solvent polarity in 15–60% ACN solvents.

**Figure 3:**
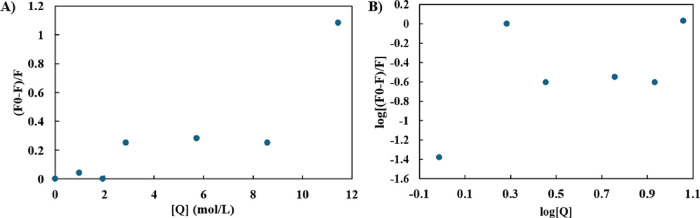
(A) Stern–Volmer plot for the quenching of BSA by ACN [Q] at different concentrations. (B) Modified Stern–Volmer plot for the binding of ACN with BSA.

The fluorescence quenching of Trp residues and a wavelength shift might result from changes in the local environment polarity around the Trp residues upon the binding of small molecules^41^ or due to the structural changes in the protein conformation.^42^ The change in fluorescence intensity due to the presence of the quencher is given by the Stern–Volmer relationship,^43^ shown in eq 1, and might be used to characterize the binding between protein and the quencher [Q] of various concentrations:
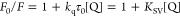
1where *k*_q_ is the quenching rate constant, and τ_0_ is the fluorophore lifetime. The product of *k*_q_ and τ_0_ is known as the Stern–Volmer constant, *K*_SV_, which shows the accessibility of the quencher to the fluorophore. The *K*_SV_, *k*_q_, and τ values were obtained from the slope of the linear trendline obtained from the plot of (*F*_0_ – *F*)/*F* against [Q] as shown below (Figure 3A). Furthermore, the binding constant (*K*_a_) and the number of binding sites (*n*) can be measured by using the intercept and the slope obtained from eq 2 and the corresponding plot (Figure 3B), respectively:
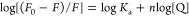
2

According to Figure 3A, considering τ_0_ as a fluorescence lifetime of BSA as 10 ns,^44^ the calculated *K*_SV_ and *k*_q_ values are 0.10 L mol^–1^ and 1 × 10^7^ L mol^–1^ s^–1^, respectively. Furthermore, a nonlinear dependence of fluorescence intensity change on the quencher concentration is observed in Figure 3B, suggesting that the fluorescence quenching of Trp residues is not linearly dependent on the concentration of ACN. In particular, while no significant quenching was observed in 5–10% ACN, a plateau with a similar quenching pattern was observed in the 15–45% ACN region. Moreover, considering 5–15% ACN conditions, two binding sites were calculated (*n* = 2.0, *K*_a_ = 0.07 mol/L), while the number of binding sites was decreased to almost one binding site in the presence of 15–60% ACN to *n* = 0.8 and *K*_a_ = 0.08 mol/L, indicating the decline of the number of BSA binding sites at decreased solvent polarity, associated with protein aggregation.

### Thioflavin T (ThT) Analysis

**Figure 4:**
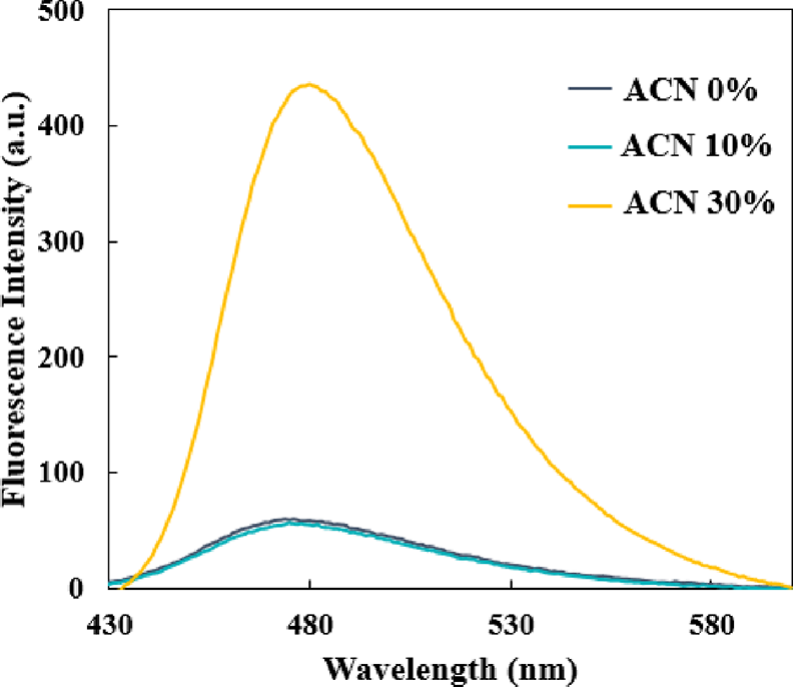
ThT analysis of BSA in 0, 10, and 30% ACN: fluorescence was recorded for samples of BSA solutions with ThT under excitation (wavelength: 410 nm).

ThT analysis was performed to investigate the protein structural rearrangements at three specific ACN concentrations of 0, 10, and 30 vol % (Figure 4). The ACN concentrations were chosen as the representative solvent compositions possessing different effects on the BSA structure, as was observed by UV spectral analyses. In particular, BSA in 60% ACN is not good for ThT fluorescence analyses because of its cloudiness due to the high denaturation of BSA. According to Figure 4, the ThT fluorescence intensity increased dramatically in 30% ACN, in comparison to 0 and 10% ACN solutions. This observation indicated that BSA was denatured, and the β-sheet content increased at a high concentration of ACN (30% ACN). Considering the low difference between the fluorescence intensity of BSA in 0 and 10% ACN, it suggested that the small amount of ACN in the 10% ACN solution did not affect the BSA structure or that ACN was almost completely removed from the solution due to its high volatility.

### FT-IR Spectra

**Figure 5:**
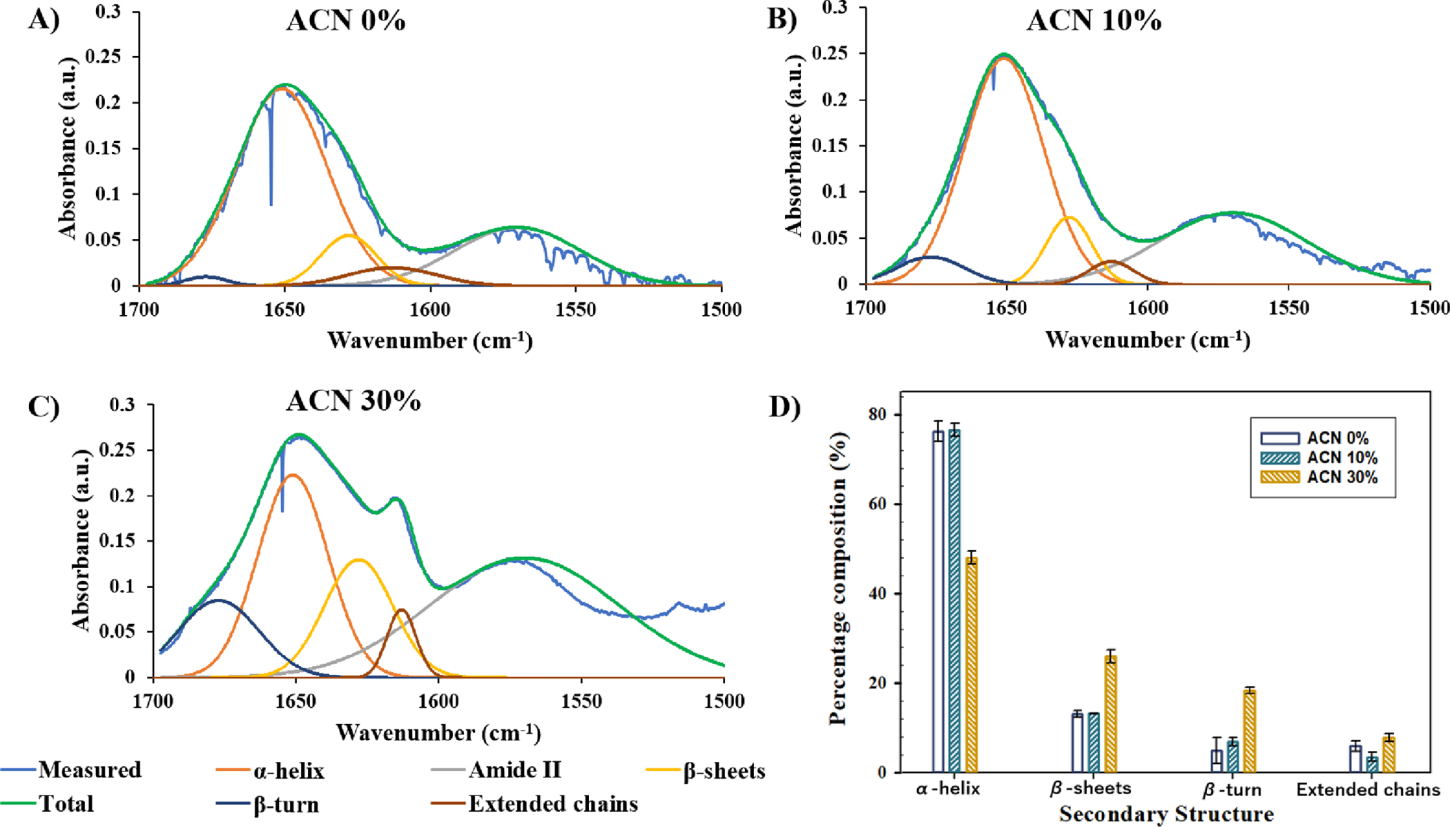
Gaussian fitting of the FT-IR spectra of BSA based on the absorption peaks of amide II = 1570 cm^–1^, intermolecular extended chains = 1613 cm^–1^, β-sheets = 1628 cm^–1^, α-helix = 1651 cm^–1^, and β-turn = 1677 cm^–1^ under different solvent conditions, (A) 0% ACN, (B) 10% ACN, and (C) 30% ACN, and (D) averaged composition of the BSA secondary structure in 0, 10, and 30% ACN.

Furthermore, FT-IR spectra were investigated for solutions of BSA in D_2_O in 0, 10, and 30% ACN (Figure 5A–C, respectively). Figure 5 shows the Gaussian fitting of the FT-IR spectra of the BSA secondary structure characterized by the following absorption peaks: amide II = 1570 cm^–1^, intermolecular extended chains = 1613 cm^–1^, β-sheet = 1628 cm^–1^, α-helix = 1651 cm^–1^, and β-turn = 1677 cm^–1^.^45^

According to Figure 5D, the major component of the BSA secondary structure is α-helix, as was indicated by the high absorbance peak at 1651 cm^–1^, in agreement with the literature.^40,46^ Moreover, consistent with the ThT analysis (Figure 4), no significant differences between secondary structures of BSA in 0 and 10% ACN were observed, with the BSA secondary structure consisting of 76.2 ± 2.32 and 76.5 ± 1.47% α-helix, 13.0 ± 0.705 and 13.2 ± 0.097% β-sheet, 4.88 ± 2.94 and 6.85 ± 0.926% β-turn, and 5.89 ± 1.26 and 3.50 ± 1.07% intermolecular extended chains in 0 and 10% ACN, respectively (Figure 5D). In comparison, as the concentration of ACN increased to 30 vol %, significant structural changes of BSA were observed. In particular, according to Figure 5C, a high absorbance peak at 1613 cm^–1^ was observed in the presence of 30% ACN, indicating the possible protein aggregation and formation of high amounts of intermolecular β-sheet aggregates and extended sheets.^47^ As shown in Figure 5D, the corresponding composition of the secondary structure of BSA in 30% ACN is α-helix = 48.0 ± 1.41%, β-sheet = 25.9 ± 1.46%, β-turn = 18.3 ± 0.751%, and intermolecular extended chains = 7.81 ± 0.960%.

### Hydrodynamic Diameter, ζ-Potential, and pH Measurements

**Figure 6:**
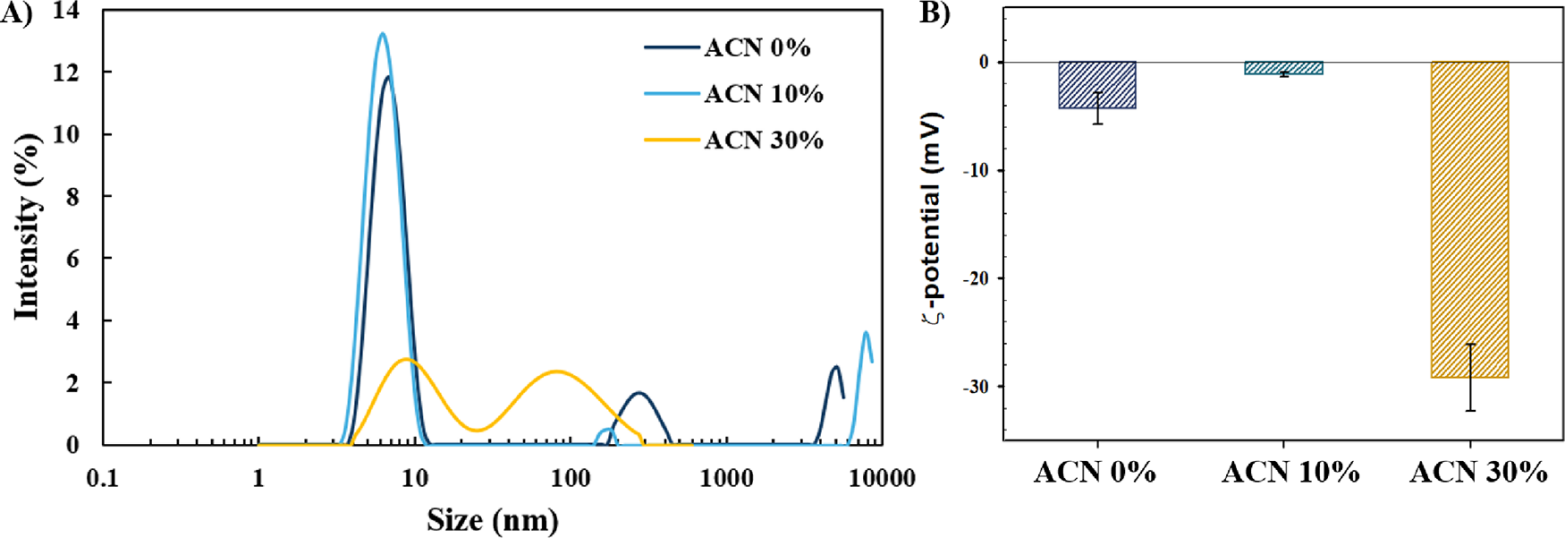
(A) Hydrodynamic diameter distribution and (B) ζ-potential of BSA (3 mg/mL) in 0, 10, and 30% ACN.

To further characterize the possible formation of protein aggregates, the hydrodynamic diameter distribution and ζ-potential were measured in the solutions with albumin (3 mg/mL) at different solvent polarities after incubation for 24 h in the presence of 0.15 M NaCl (Figure 6). The pH values of the incubated solutions with BSA in 0, 10, and 30% ACN were 6.58, 6.67, and 6.68, respectively, indicating the neutral pH conditions in the solutions under study.

According to Figure 6A, the hydrodynamic diameter distribution of protein in 0 and 10% ACN has similar maximum intensity values at a size of 7 nm (P.I. = 0.221 and 0.284 in 0 and 10% ACN, respectively). In comparison, in 30% ACN, the hydrodynamic diameter distribution is significantly different with double maximum peaks at the sizes of 9 and 80–90 nm and a wider size distribution (P.I. = 0.303), indicating the formation of protein aggregates at decreased solvent polarity, consistent with our observations from ThT and FT-IR analyses. Considering the formation of protein aggregates at decreased solvent polarity, our results are consistent with the results of Pabbathi et al.,^24^ who observed the increase in the hydrodynamic radius of BSA with increasing DMSO concentration, suggesting the protein unfolding and formation of BSA aggregates.

Interestingly, according to Figure 6B, the ζ-potential values were significantly decreased in the presence of ACN at high concentration, from the average value of −4.62 mV in 0% ACN to −29.17 mV in 30% ACN. While the enhanced electrostatic repulsions are expected to reduce the formation of protein aggregates, our experiments showed the formation of protein aggregates with anionic surface charge, bringing new insights into the mechanism of protein aggregation at decreased solvent polarity. In particular, it might be suggested that at decreased solvent polarity and increased solvent hydrophobicity in 30% ACN, the hydrophobic domain of albumin becomes exposed to the solvent and facilitates protein aggregation via hydrophobic interactions, resulting in the formation of protein aggregates by overcoming the electrostatic interactions, as will be discussed further from the results of the MD simulations.

### MD Simulations

**Figure 7:**
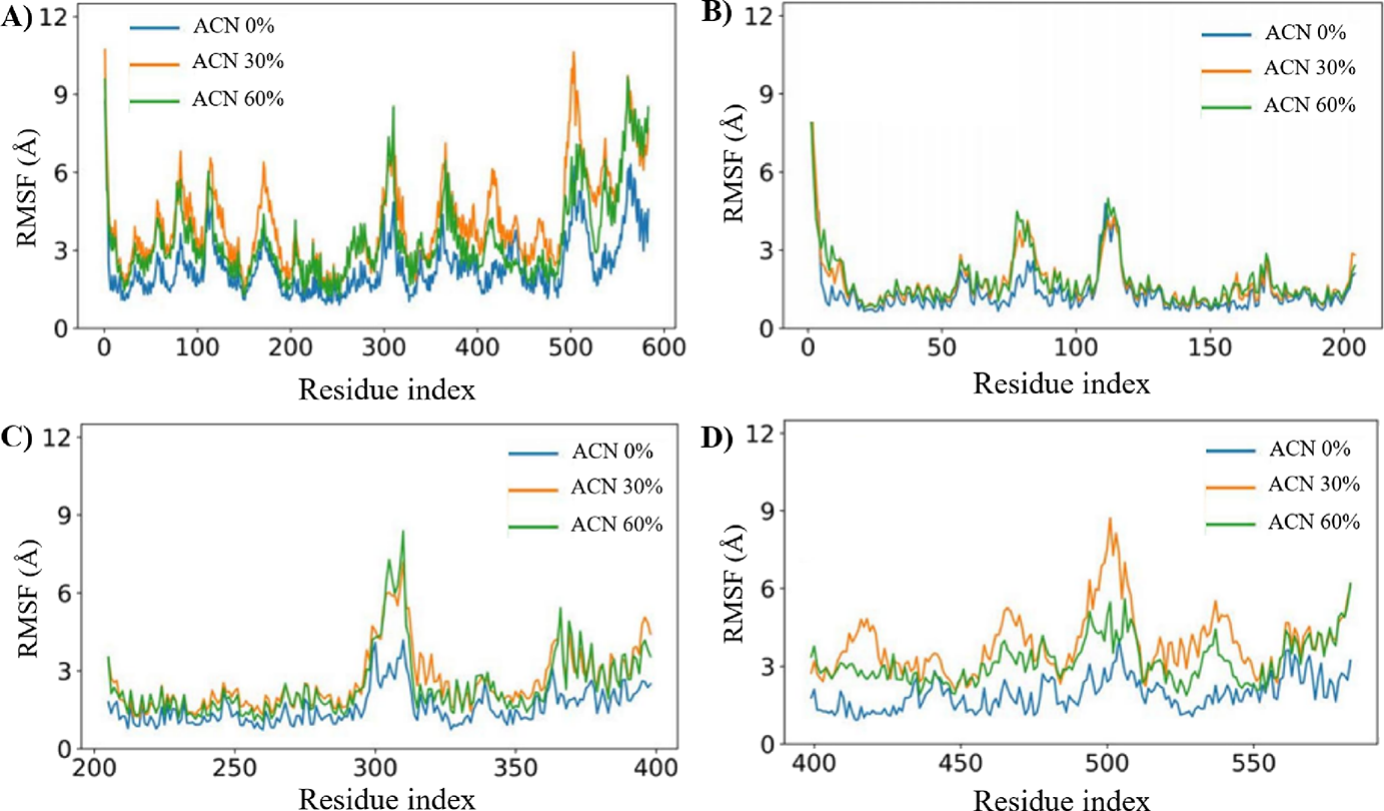
RMSF of albumin residues in solutions with different polarities: (A) BSA structure (1–583 amino acid residues), (B) domain I (1–204 amino acid residues), (C) domain II (205–398 amino acid residues), and (D) domain III (399–583 amino acid residues).

MD simulations were further performed to detect the unstable regions of albumin in water (0% ACN), 30% ACN, and 60% ACN under different solvent polarities. The root-mean-square fluctuations (RMSFs) of the BSA structure were calculated from the MD trajectories, as shown in Figure 7A. In addition, the RMSF values were calculated for each BSA domain separately (Figure 7B–D), eliminating the effect of interdomain fluctuations.

According to Figure 7A, the albumin structure has comparatively smaller RMSF values (up to 6 Å) in water than in the solutions containing ACN. This observation indicates that the BSA structure is more rigid and stable in water than in the presence of ACN. On the other hand, the data for solutions containing ACN showed that the RMSF value was higher on average, with multiple locations showing a high RMSF value of over 10 Å. Overall, the RMSF values calculated over the entire protein structure (583 amino acids) are relatively large due to the relative motion of amino acids between domains (Figure 7A). According to Figure 7B–D, domain I and domain II are comparatively more stable than domain III. The addition of ACN increases the fluctuations in the positions of the amino acids in all three domains.

According to Figure 7B, in domain I, the maximum RMSF values of the amino acid region located around residue Leu-80 in subdomain IA increased from 2.5 Å in water to 4.1 and 4.5 Å in the 30 and 60% ACN conditions, respectively. In contrast, considering the hydrophilic subdomain IB (108–196 a.a.), no significant changes in the RMSF values were observed with the addition of ACN. Moreover, the fluctuations around Trp-134 are not so large in the presence of ACN, indicating that Trp-134 did not contribute to the shift of UV. In domain II, the presence of ACN destabilizes the conformation around the hydrophobic region (between Leu-301 and Ala-309) and the region between Cys-360 and Tyr-400 (Figure 5C). In particular, the increased maximum RMSF values were observed in domain II from 4.0 Å in 0% ACN to 7.3 and 8.4 Å in 30 and 60% ACN conditions, respectively. Similarly, according to Figure 7D, the RMSF fluctuations of domain III are significantly larger in the presence of ACN (RMSF values in the range of 1.9–8.8 Å) than in water (RMSF values up to 4.1 Å). In particular, high RMSF values were observed in the hydrophobic subdomain IIIA, a drug-binding site (Sudlow II site), indicating a more prominent effect of ACN on the hydrophobic regions of albumin.

**Figure 8:**
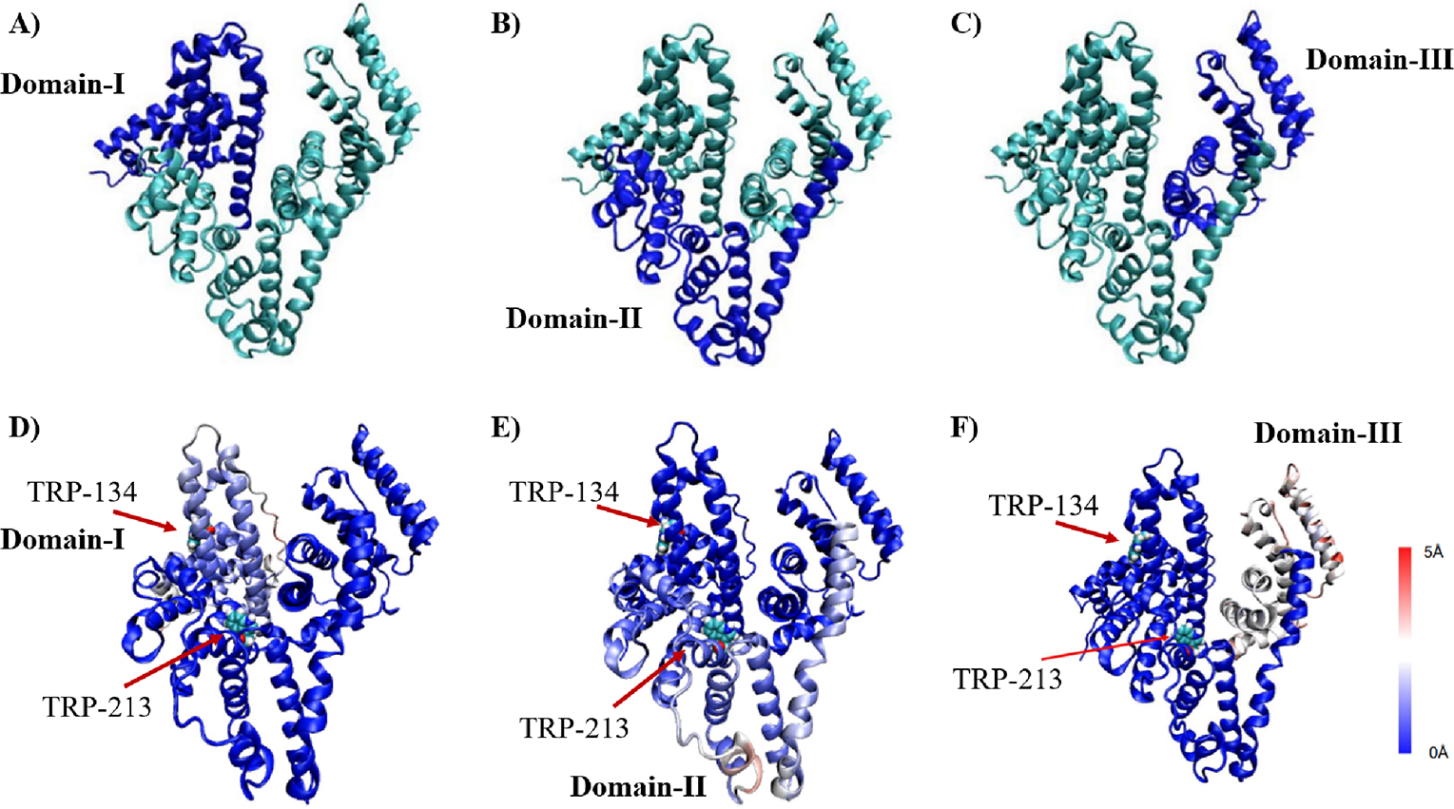
(A–C) Visualized and highlighted BSA domains (in dark blue): (A) domain I (1–204 amino acid residues), (B) domain II (205–398 amino acid residues), and (C) domain III (399–583 amino acid residues); (D–F) visualized RMSF distribution within a particular domain in the 60% ACN solvent: (D) domain I, (E) domain II, and (F) domain III.

The RMSF values observed from the simulations with 60% ACN were further visualized in the BSA monomer structure for each domain separately (Figure 8). Figure 8A–C highlights the position of domains I–III, colored blue, and Figure 8D–F shows each domain colored with the magnitude of the fluctuation. Domains not of interest in Figure 8D–F are colored blue, identical with the 0 Å fluctuations. According to Figure 8D–F, the high-RMSF regions, colored red and white, are mostly located at the edges of albumin interacting with the solvent. Trp-213 is within the domain II region, but the fluctuations around Trp-213 in domain II are small and do not appear to be more exposed to the solvent due to the mixing of ACN. However, Trp-213 is in the proximity of domain III, which becomes highly unstable in solutions containing ACN (Figure 7D). High fluctuations in the positions of amino acids observed near the hydrophobic pocket with the Trp-213 residue (Figure 8F) could be associated with the destabilization of the hydrophobic pocket and increased UV intensity observed in the experiments (Figure 2).

**Figure 9:**
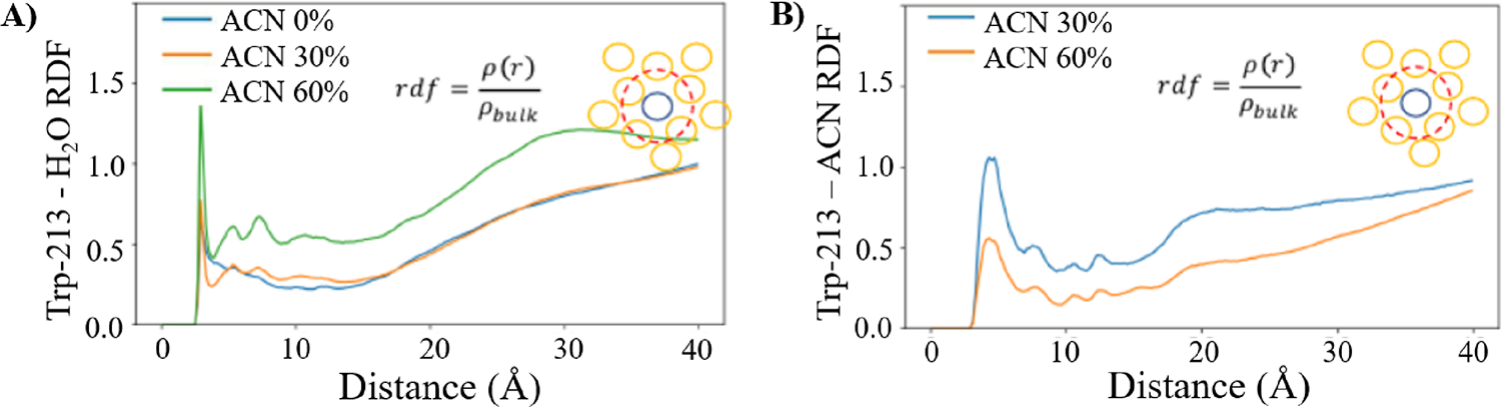
RDFs between (A) Trp-213 (N atom) and water and (B) Trp-213 (N atom) and ACN.

The solvent accessibility of Trp residues was further studied via radial distribution function (RDF) analysis (Figure 9). According to Figure 9A, the interactions between solvent-accessible Trp-213 and water significantly varied in 60% ACN with low polarity. Comparing the first maximum RDF peaks at the distance of 3 Å, known as a first hydration shell, the peak values were around 1.4 in 60% ACN and around 0.7 in 0 and 30% ACN (Figure 9A). Next, the interactions between Trp-213 and ACN were studied in 30 and 60% ACN (Figure 9B). Similarly, the results showed the presence of ACN molecules near the hydrophobic pocket with Trp-213 residue. In particular, maximum RDF peak values of around 1.0 and 0.5 were observed at a 5 Å distance (first hydration shell) in 30 and 60% ACN, respectively.

Although the RDF of ACN around Trp-213 was comparatively high under both 30 and 60% ACN conditions, the results show enhanced interactions between Trp-213 and water molecules in 60% ACN. As shown by the RMSF analyses (Figures 7 and 8), ACN could disturb the hydrophobic pocket through domain III, which was highly unstable in solutions containing ACN, resulting in the intrusion of water molecules into the hydrophobic pocket with Trp-213 in 60% ACN (Figure 9A). This observation is correlated with inducing the changes in the BSA secondary structure at reduced solvent polarity, consistent with the results of our spectroscopical analyses. Moreover, under 30% ACN conditions with moderate solvent polarity, the interactions between Trp-213 and water were not significantly increased, suggesting that the local microenvironment around Trp-213 would be affected by ACN molecules located in the hydration shell (Figure 9B). Overall, while the intrusion of both ACN and water molecules into the BSA hydrophobic pocket was observed in the presence of ACN (30 and 60 vol %), the high fluctuations of amino acid residues near the hydrophobic pocket with Trp-213 residue (Figure 8F) in the BSA conformation in 60% ACN indicate the importance of solvent polarity for the stability of the protein conformation.

While previous experimental studies showed the conformational changes in the microenvironment around Trp residues with a reduction of the local polarity in the presence of DMSO,^24^ which was in agreement with the results of our experiments (Figure 1), the results of our MD simulations showed that the presence of ACN enhanced the fluctuations in the positions around hydrophobic domain III and affected the microenvironment around Trp-213, with no significant effect on the Trp-134 residue. Considering that subdomain IIIA with the drug-binding site (Sudlow II site) of BSA was affected by ACN more significantly, as was revealed by the RMSF analysis, the solvent polarity should be controlled in DDS with proteins to avoid the structural modifications of the drug-loading site. Although any drug binding to albumin was not studied in this work, we will investigate the binding in the presence of ACN in future plans.

## Conclusions

The present study investigated the effect of solvent polarity, controlled by adding ACN in water, on the conformation of BSA by optical analysis. An increase in the level of ACN in an aqueous solution of BSA shifted the UV fluorescence wavelength and decreased its intensity. In addition, ThT, FT-IR, and DLS analyses showed the protein aggregation and formation of high amounts of intermolecular β-sheet aggregates and extended sheets at high concentrations of ACN (30 vol %). Furthermore, MD simulations suggested that ACN destabilized the water-dependent conformation around the indole ring of Trp residues of BSA. Such conformational changes without any amino acid mutations of the protein may open new possibilities for DDS applications of proteins by loading drug molecules. Overall, the insights gained from this research have significant implications for the design and optimization of protein-based nanocarriers for various biomedical applications.

## Accession Codes

Bovine serum albumin: UniProtKB P02769.
